# Genistein mediates the selective radiosensitizing effect in NSCLC A549 cells via inhibiting methylation of the keap1 gene promoter region

**DOI:** 10.18632/oncotarget.8403

**Published:** 2016-03-26

**Authors:** Xiongxiong Liu, Chao Sun, Bingtao Liu, Xiaodong Jin, Ping Li, Xiaogang Zheng, Ting Zhao, Feifei Li, Qiang Li

**Affiliations:** ^1^ Institute of Modern Physics, Chinese Academy of Sciences, Lanzhou 730000, China; ^2^ Key Laboratory of Heavy Ion Radiation Biology and Medicine, Chinese Academy of Sciences, Lanzhou 730000, China; ^3^ Key Laboratory of Basic Research on Heavy Ion Radiation Application in Medicine, Gansu Province, Lanzhou 730000, China; ^4^ University of Chinese Academy of Sciences, Beijing 100049, China

**Keywords:** genistein, selective radiosensitivity, Keap1/Nrf2, methylation, oxidative stress

## Abstract

Non-small cell lung cancer (NSCLC) cells often possess a hypermethylated Keap1 promoter, which decreases Keap1 mRNA and protein expression levels, thus impairing the Nrf2-Keap1 pathway and thereby leading to chemo- or radio-resistance. In this study, we showed that genistein selectively exhibited a radiosensitizing effect on NSCLC A549 cells but not on normal lung fibroblast MRC-5 cells. Genistein caused oxidative stress in A549 cells rather than MRC-5 cells, as determined by the oxidation of the ROS-sensitive probe DCFH-DA and oxidative damage marked by MDA, PCO or 8-OHdG content. In A549 instead of MRC-5 cells, genistein reduced the level of methylation in the Keap1 promoter region, leading to an increased mRNA expression, thus effectively inhibited the transcription of Nrf2 to the nucleus, which suppressed the Nrf2-dependent antioxidant and resulted in the upregulation of ROS. Importantly, when combined with radiation, genistein further increased the ROS levels in A549 cells whereas decreasing the radiation-induced oxidative stress in MRC-5 cells, possibly via increasing the expression levels of Nrf2, GSH and HO-1. Moreover, radiation combined with genistein significantly increased cell apoptosis in A549 but not MRC-5 cells. Together, the results herein show that the intrinsic difference in the redox status of A549 and MRC-5 cells could be the target for genistein to selectively sensitize A549 cells to radiation, thereby leading to an increase in radiosensitivity for A549 cells.

## INTRODUCTION

Selectively killing cancer cells without harming normal cells is a fundamental challenge in cancer therapy. Elevated oxidative stress has been found in many types of cancer cells, due in part to their metabolic rates, when compared with their normal cell counterparts [1]. To adapt to this oxidative status, many tumor cells possess strong antioxidant defense mechanisms to counteract excessive reactive oxygen species (ROS), maintain redox status, suppress apoptosis and promote growth [2]. Thus, selectively impairing the antioxidant defense system can make tumor cells have high constitutive oxidative stress levels, leading to cell death, whereas normal cells may still maintain redox homeostasis through adaptive responses.

The nuclear factor-erythroid 2-related factor 2 (Nrf2)/Kelch-like ECH-associated protein 1 (Keap1) system plays an important role in the cellular defense against oxidative stress [3]. Under normal physiological conditions, Nrf2 forms an inactive complex with a negative regulator, Keap1. Oxidation or phosphorylation of cysteine residues facilitates the dissociation of Nrf2 from Keap1, with subsequent translocation to the nucleus [4, 5], where it binds to the antioxidant response element (ARE). The ARE is a cis-acting regulatory element of genes encoding phase II detoxification enzymes and antioxidant proteins, such as NAD(P)H quinone oxidoreductase-1 (NQO-1), heme oxygenase-1 (HO-1), to protect against oxidative damage [6]. So, if the activity of Keap1 is impaired, which can lead to full Nrf2 activation, cancer cells may acquire a protective mechanism against the surrounding microenvironment [7, 8]. Numerous studies have shown that the Nrf2/Keap1 system can protect normal cells from exogenous ROS, but promotes the death of cancer cells under deleterious conditions [9, 10].

DNA methylation, which was the first epigenetic mechanism recognized and has been most extensively studied, primarily occurs at cytosines that precede guanines in dinucleotide CpG sites, via addition of a methyl group to the 5′ position of the cytosine ring to form 5-methylcytosine [11, 12]. DNA methylation plays important roles in carcinogenesis and the regulation of radiosensitivity of cancer cells [13–15]. Site-specific methylation within promoters has, in many cases, been associated with the transcriptional silencing of specifically regulated genes [16]. Keap1 was confirmed as a repressor of Nrf2. Wang *et al.* reported that the promoter region of Keap1 is aberrantly hypermethylated and Keap1 mRNA expression levels are low in some lung cancer cell lines and lung cancer tissues; however, Keap1 is highly expressed in BEAS-2B human normal bronchial epithelial cells [17]. Genistein is a natural isoflavone with many biological activities. Xie *et al.* suggested that genistein has a significant inhibitory effect on global DNA methylation levels in breast cancer cells [18]. In addition, several studies [19, 20] have showed that genistein can reverse hypermethylation and reactivate several TSGs in cancer cells. However, whether genistein regulates the methylation level of the Keap1 promoter region and the subsequent expression of Keap1 have not been elucidated yet.

The aim of this study was to investigate how genistein differently modulates the intracellular redox status in human non-small cell lung cancer A549 cells and human normal lung fibroblast MRC-5 cells, identify the targets of genistein in the Nrf2-Keap1 pathway, and evaluate the radiosensitizing effect of genistein on A549 cells.

## RESULTS

### The radiosensitizing effect of genistein was selective for A549 cells instead of MRC-5 cells

**Figure 1:**
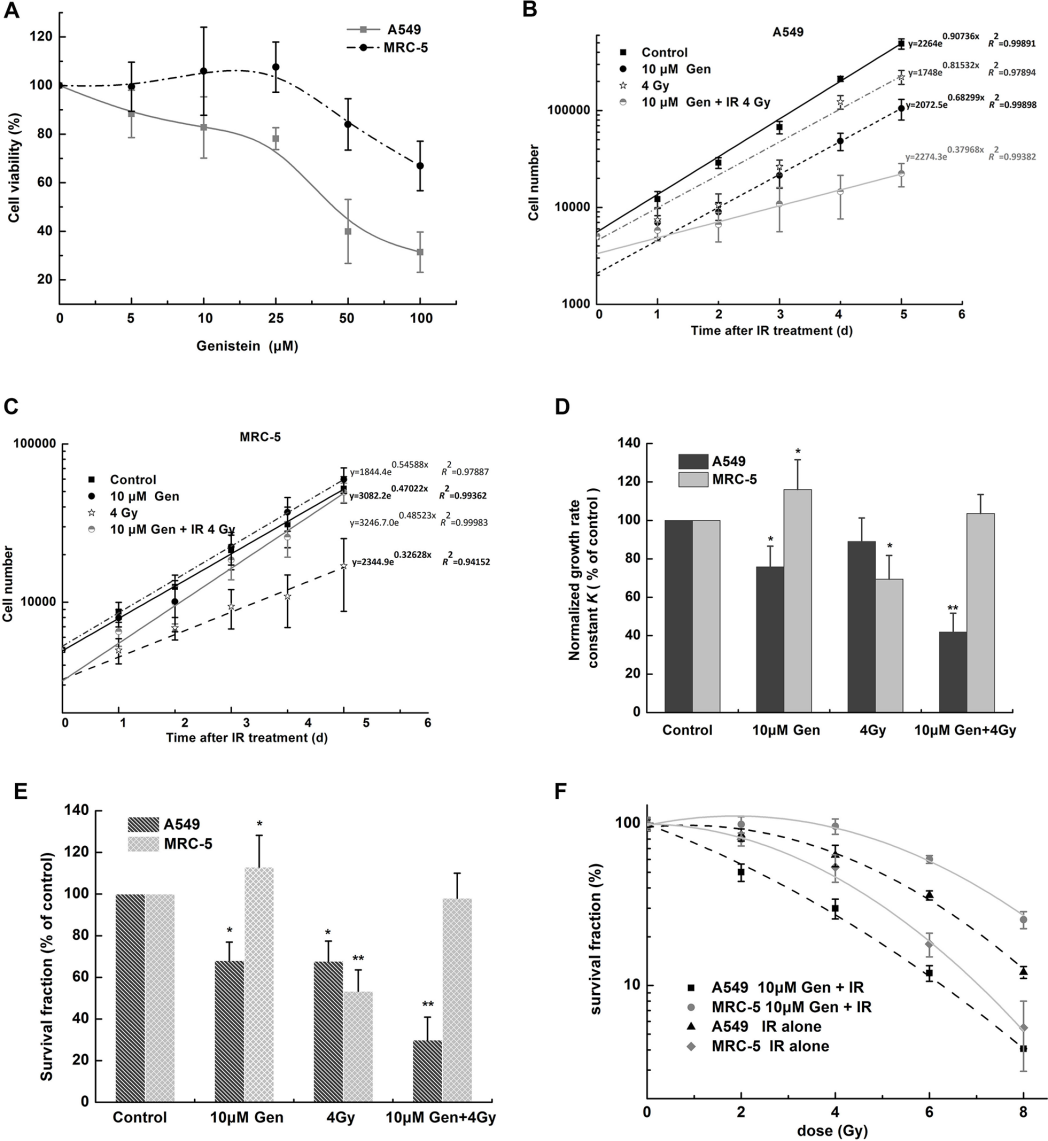
The radiosensitizing effect of genistein was selective for A549 cells but not for MRC-5 cells (**A**) MTT assay. Cell viability was measured after 48 h of genistein treatment. (**B**) and (**C**) cell growth curves. Cell numbers were plotted on a log-linear scale. The data points of the first 2 days were excluded in the data fitting. Equations derived from the exponential growth curve fit [Y = start × exp (*k* × *X*)] are shown. Cell number began at Y = start and increased exponentially with a rate constant *k*. (**D**) comparison of the growth rate constant in A549 and MRC-5 cells after genistein and radiation co-treatment. The growth rate constant (*K*) was obtained from the exponential growth curve fit in (B and C), then normalized to the control group for each cell line (growth rate constant *K* = *k*_(sample)_/*k*_(control)_), respectively. (**E**) and (**F**) show the data from the clonogenic survival assay. *p < 0.05, **p < 0.01 *vs*. the control group. Gen, genistein; IR, irradiation.

Firstly, we performed a MTT assay under the growth condition to provide cell viability. MRC-5 cells were found to be more resistant to the genistein-induced cytotoxicity compared with A549 cells (Figure 1A). The subcytotoxic dose of genistein (10 μM) was chosen to study the combined effect of genistein and radiation on cell radiosensitivity. Comparisons of the growth curves and survival fractions for the two cell lines indicated a selectively radiosensitizing effect of genistein on A549 cells. For example, in Figure 1D, genistein alone decreased the number of A549 cells in growth rate by 24.2 ± 1.5%, but increased the number of MRC-5 cells in growth rate by 16.0 ± 1.3%. Radiation (4 Gy) decreased the cell growth rate by 11.0 ± 1.0% in A549 cells and by 31.6 ± 2.9% in MRC-5 cells. Interestingly, the growth rate in the combined treatment group was almost the same as the control group for MRC-5 cells, but decreased by 59.2 ± 3.9% in A549 cells. Similar results were derived from the clonogenic survival data as shown in Figure 1E.

To further investigate if genistein selectively enhanced the radiosensitivity of A549 cells, the clonogenic survival curves were acquired. As illustrated in Figure 1F, genistein enhanced the radiosensitivity of A549 cells with a radiation enhancement ratio of 1.66 at 50% cell survival (IC_50_); however, genistein had a radio-protective effect on MRC-5 cells.

### Genistein aggravated the oxidative stress and oxidative damage induced by radiation in A549 cells but not in MRC-5 cells

**Figure 2:**
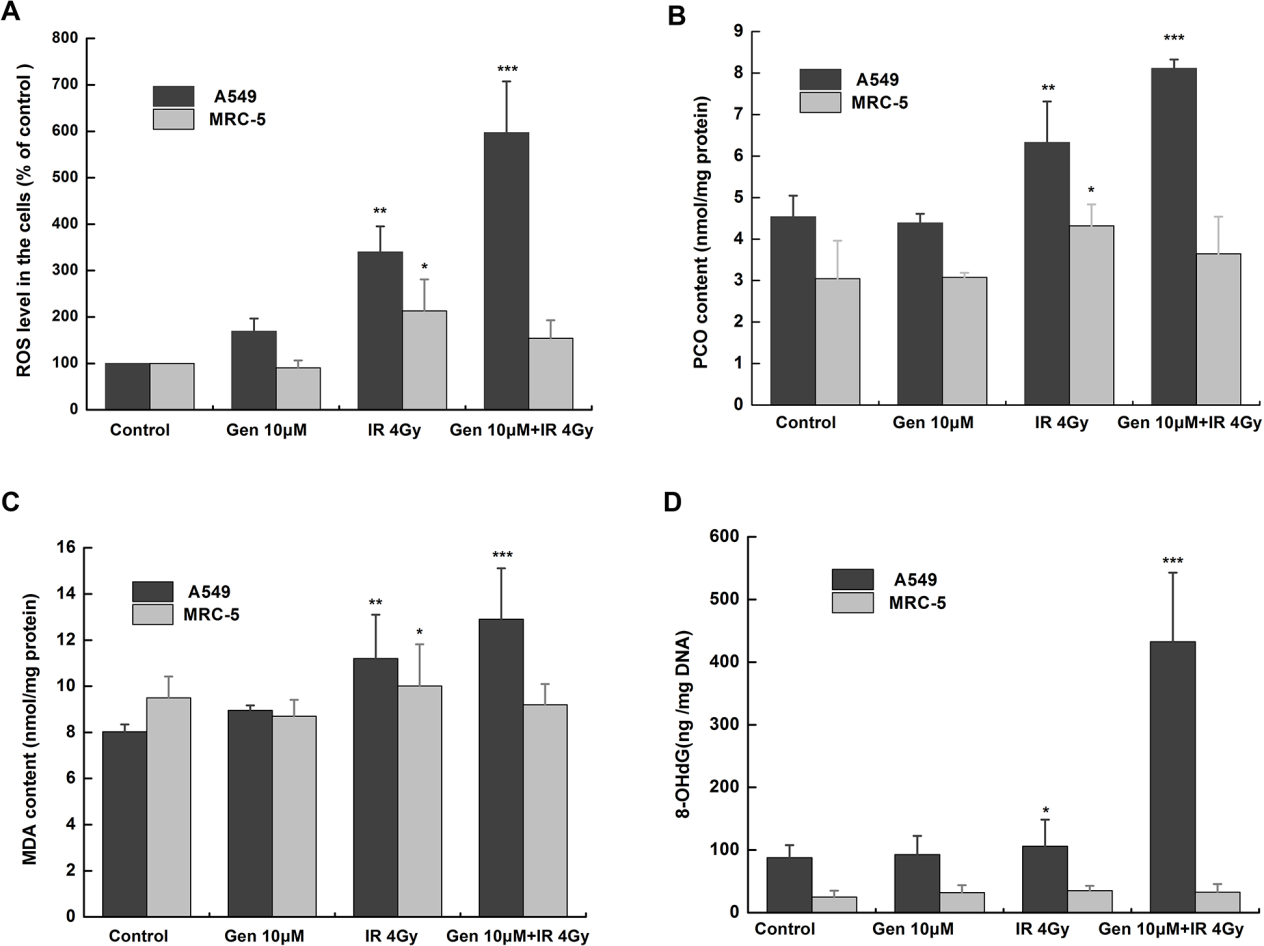
Genistein induced oxidative stress and oxidative damage in A549 rather than in MRC-5 cells (**A**) DCFH-DA assay. Cells were treated with 10 μM genistein for 48 h then with or without irradiation; (**B**) PCO; (**C**) MDA and (**D**) 8-OHdG levels. *p < 0.05, **p < 0.01, ***p < 0.001 *vs*. the control group.

We then explored potential determinants for the selectivity of the effect of genistein. Oxidative stress is the major mechanism for radiation-induced cancer cell death. As shown in Figure 2A, the radiation alone significantly increased the ROS levels both in A549 cells (*p* < 0.01) and in MRC-5 cells (*p* < 0.05). However, genistein alone elicited an increase of the ROS level in A549 cells rather than in MRC-5 cells. When combined with radiation, genistein further increased the cellular ROS level in A549 cells, thereby promoting the cell-killing effect. Importantly, in MRC-5 cells, genistein decreased the radiation-induced ROS level, suggesting an antioxidant response by genistein.

Oxidative damage to proteins and lipids were measured as PCO and MDA [21, 22], respectively. The results are shown in Figure 2B and 2C. Consistent with the ROS production, 4 Gy radiation significantly increased the PCO and MDA contents both in A549 cells (*p* < 0.01) and in MRC-5 cells (*p* < 0.05). However, in the combined treatment group, the PCO and MDA contents increased significantly (*p* < 0.001) in A549 cells but not in MRC-5 cells. Simultaneously, DNA oxidative damage was studied by quantifying the levels of modified base 8-OHdG in both cell lines [23], and the results are shown in Figure 2D. Clearly, the modified base level of the combined treatment group increased significantly in A549 cells in comparison with the control group (*p* < 0.001).

### Genistein decreased reduced GSH in A549 cells but increased it in MRC-5 cells

**Figure 3:**
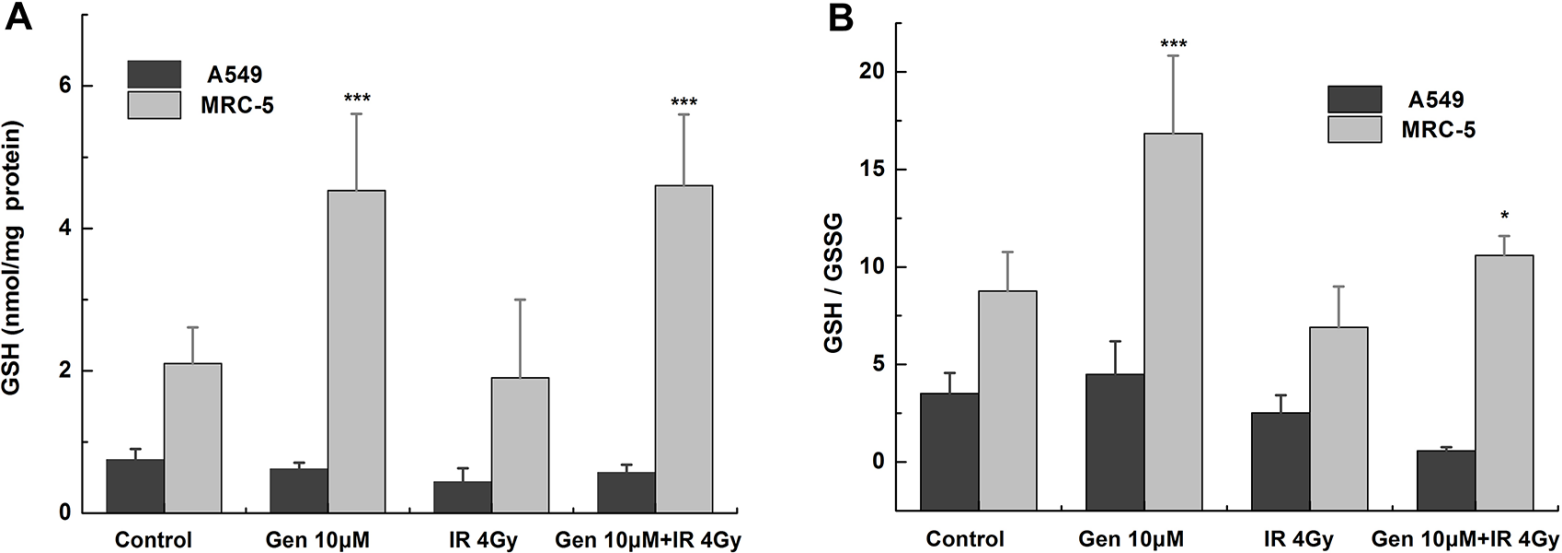
Levels of GSH and GGH/GSSG ratio Cells were treated with 10 μM genistein for 48 h then with or without irradiation. (**A**) GSH; (**B**) the ratio of GSH to GSSG. *p < 0.05, ***p < 0.001 *vs.* the control group.

As shown in Figure 3A, compared with the control group, the total GSH level was hardly changed in A549 cells but significantly increased in MRC-5 cells (*p* < 0.001) by genistein treatment. The reduced GSH/GSSG ratio was not significantly changed by genistein in A549 cells but increased by a factor of 1.92 in MRC-5 cells (Figure 3B), which may lead to the protective effect against the radiation-induced oxidative stress observed through the DCFH-DA assay. The ratio of GSH to GSSG was also significantly enhanced by genistein combined with X-ray treatment in MRC-5 cells (*p* < 0.05) (Figure 3B). Taken together, these results suggest a reducing type of redox environment in MRC-5 cells compared to that of A549 cells, and this could be the reason for the enhanced tolerability of MRC-5 cells against radiation exposure.

### Genistein induced the expression of Keap1 via its promoter CpG island demethylation in A549 cells

**Figure 4:**
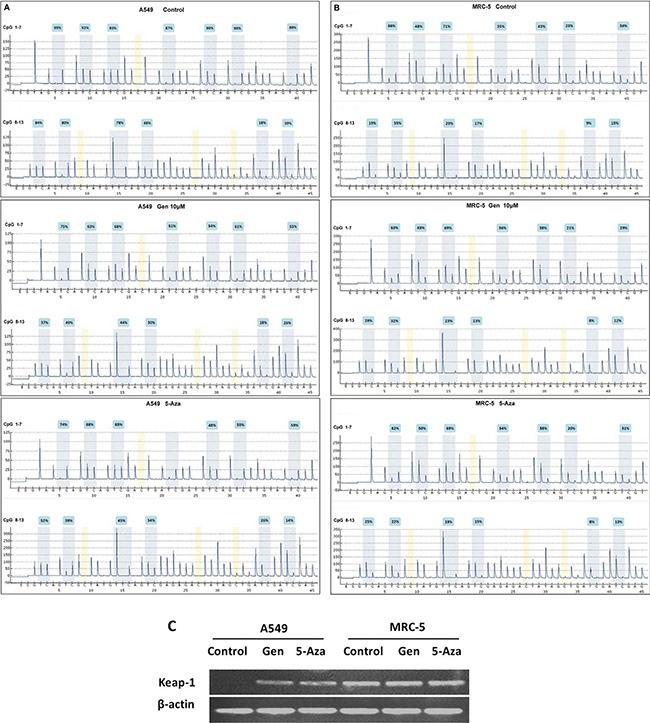
Genistein induced the Keap1 mRNA expression via demethylation of its promoter CpG islands in A549 but not in MRC-5 cells (**A**) the Keap1 mRNA expression was analyzed by RT-PCR assay. (**B**) (A549 cells) and (**C**) (MRC-5 cells) show the methylation levels of the Keap1 promoter CpG islands, which were analyzed by pyrosequencing assay.

In the present study, the bisulfite pyrosequencing assay could be used to examine the DNA methylation level of 13 CpG sites in the Keap1 promoter, which is almost identical to the regions examined by Wang *et al.* [17]. As shown in Figure 4A and 4B, two sequencing primers were used for the pyrosequencing reaction, with the first reaction examining seven CpG sites (1–7), while the second reaction examined six additional CpG sites (8–13). In A549 cells, the mean methylation level was 76% in the control group, but decreased to 48% in the genistein treated group. To further determine whether methylation level of the Keap1 promoter was regulated by genistein, we treated cells with 5-Aza, a DNA methylation transferase (DNMTs) inhibitor [24, 25]. The 5-Aza treatment reduced the mean methylation level of the Keap1 promoter genes to 46% (Table 1).

**Table 1:** The methylation data for each CpG site within all tested region of the Keap1 promoter

Sample	CpG site														
	1	2	3	4	5	6	7	8	9	10	11	12	13	Ave.
**A1**	95%	92%	93%	87%	90%	93%	88%	84%	80%	78%	48%	18%	39%	76%
**A2**	75%	63%	68%	51%	54%	61%	55%	37%	49%	44%	30%	18%	25%	48%
**A3**	74%	68%	65%	48%	55%	59%	52%	39%	45%	34%	26%	14%	21%	46%
**M1**	66%	48%	71%	35%	43%	23%	34%	19%	35%	29%	17%	9%	15%	32%
**M2**	63%	43%	69%	36%	38%	21%	29%	28%	32%	23%	13%	8%	12%	32%
**M3**	62%	50%	69%	34%	38%	20%	31%	25%	22%	19%	15%	8%	13%	31%

A1, A2, A3 represent A549 cells. M1, M2, M3 represent MRC-5 cells. A1, M1 were the controls; A2, M2 were treated with 10 μM genistein for 48 h; A3, M3 were treated with 10 μM 5-Aza for five days.

Hypermethylation in the gene promoter may affect its expression. Therefore, we evaluated the Keap1 mRNA levels in the two cell lines. Figure 4C shows that both genistein and 5-Aza induced re-expression of the methylated Keap1 genes through demethylation in A549 cells. However, no Keap1 mRNA expression changes were detected in MRC-5 cells.

### Genistein induced nuclear accumulation of Nrf2 in MRC-5 cells but not in A549 cells

**Figure 5:**
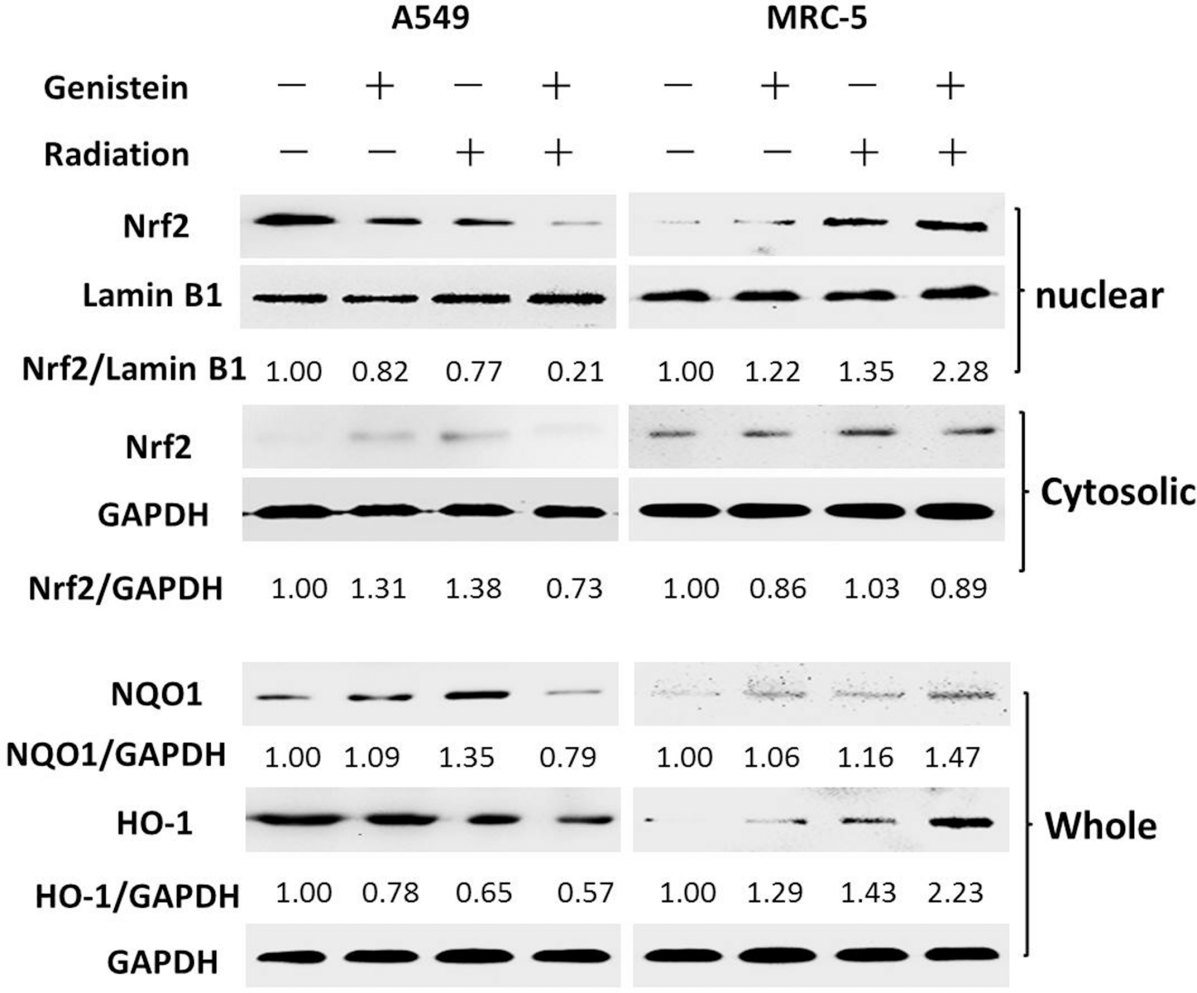
Effects of genistein on Nrf2 accumulation in the nucleus and the expression of NQO1 and HO-1 in whole protein extract

Figure 5 shows that Nrf2 clearly accumulated in the nuclear fraction of MRC-5 cells in the genistein combined with X-ray treatment group. On the contrary, in A549 cells, the expression of Nrf2 in the nuclear fraction was reduced significantly by the co-treatment. Whereas, the Nrf2 protein levels in cytoplasmic fraction were basically equivalent in the two cell lines. Moreover, the expression of antioxidant gene NQO1 and HO-1 were either activated or inhibited by Nrf2. Herein, we found that NQO1 protein expression was significantly inhibited by the genistein combined with X-ray treatment in A549 cells. Surprisingly, HO-1 protein was obviously activated by the combined treatment in MRC-5 cells.

### Genistein increased radiation-induced apoptosis in A549 cells but not in MRC-5 cells

**Figure 6:**
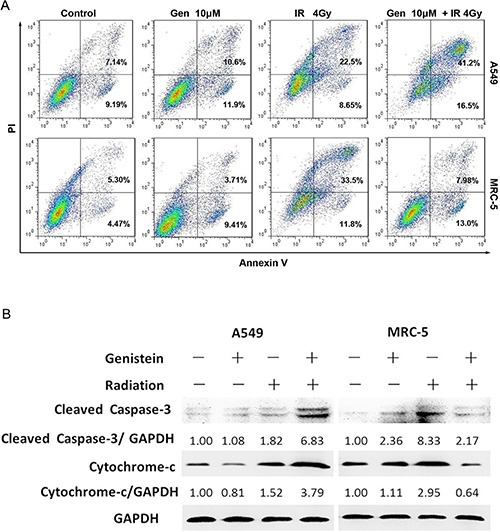
Effects of genistein on cell apoptosis and the changes in regulatory proteins induced by genistein or X-ray radiation in A549 and MRC-5 cells, respectively (**A**) Apoptosis was quantified by the combined staining of Annexin V and PI, and fluorescence was analyzed using flow cytometry. (**B**) The Caspase-3 and cytochrome c expressions were assessed by means of western blot.

To investigate the radiosensitizing effect of genistein on A549 cells, apoptosis of cells was examined by the combined staining of Annexin V and PI. In the combined treatment group, the apoptotic rate in A549 cells was approximately 56.5 ± 3.3% (early apoptosis, 41.6 ± 1.5%; late apoptosis, 15.0 ± 1.2%), in contrast to approximately 30.8 ±2.9% (early apoptosis, 21.9 ± 1.3%; late apoptosis, 8.85 ± 1.7%) in the radiation alone group (Figure 6A). However, no significant difference in apoptosis level was detected in MRC-5 cells in the co-treatment group compared with the control group (Figure 6A).

To further examine the mechanisms of apoptosis, the apoptosis-related proteins were detected by western blot. As shown in Figure 6B, the combined treatment resulted in cleavage of Caspase-3 in A549 cells but not in MRC-5 cells. Moreover, by the co-treatment, Cytochrome c levels increased in the cytoplasmic fraction in A549 cells rather than in MRC-5 cells.

## DISCUSSION

A number of studies have reported that genistein has radiosensitizing effects on cancer cells [26, 27]. The present study confirms these findings in A549 cells and further shows that the radiosensitizing effect of genistein was selective for A549 cells but not for MRC-5 cells (Figure 1). Our results also indicate that genistein protected the irradiated MRC-5 cells, because the cell growth rate (Figure 1D) and survival fractions (Figure 1E and 1F) after irradiation were restored to the untreated control level when combined with genistein treatment.

Persistent elevation of ROS can directly or indirectly disturb physiological functions of many cellular macromolecules such as DNA, proteins and lipids [28]. In the present study, we found that genistein selectively enhanced the level of ROS and aggravated the oxidative damages induced by radiation in A549 cells instead of MRC-5 cells (Figure 2A). Once suffering from oxidative stress, induction of a family of antioxidant/detoxification enzymes that enhance the cellular ROS-scavenging capacity is a key element in the maintenance of cellular redox homeostasis and in reducing oxidative damage [29]. One of the most versatile protectors of such antioxidant is GSH. Figure 3A shows A549 cells had low levels of GSH and GSH to GSSG ratio in both the basal and inducible groups; but in MRC-5 cells, due to high GSH level and GSH to GSSG ratio (Figure 3B) induced by genistein, the cellular resistance to radiation was elicited. Furthermore, we observed accumulation of NQO1 and HO-1 in MRC-5 cells but not in A549 cells (Figure 5), indicating a good antioxidant system in MRC-5 cells.

Previous studies have reported that Nrf2 is a transcription factor which is involved in the transcription of the antioxidant enzymes [30]. Therefore, we hypothesized that the Nrf2 which controls the antioxidant enzymes may play an important role in determining the radiosensitivity. Studies challenging the molecular basis of the Nrf2/Keap1 system functions are now critically important to improve translational studies of the system [31].

In A549 cells, we observed the frequent hypermethylation of the CpG islands in the promoter region of Keap1 and the reduced levels of Keap1 mRNA expression in the control group, but the hypomethylation of the CpG islands in the promoter region and the restoration of Keap1 expression by treatment with genistein. Xie *et al.* have suggested that genistein is a new DNMT inhibitor by using molecular modeling and methylation-related experiments, indicating that genistein exerts its inhibitory effect on DNMT1 function by blocking the entry of the key nucleotide, cytosine, to its active site and thus preventing methylation [18]. In the present study, the recovery of Keap1 expression by treatment with 5-Aza also strongly suggests that the Keap1 down-regulation might be due to the aberrant hypermethylation in the Keap1 promoter (Figure 4C). Moreover, our results indicate that genistein effectively induced the Keap1 expression, which inhibited the translocation of Nrf2 to the nucleus (Figure 5). Consequently, the expression of the Nrf2 downstream genes was reduced, and subsequently the Nrf2-dependent antioxidant system was suppressed. The amount of newly formed ROS was greater than those resulting from metabolism, thereby leading to oxidative stress eventually.

However, the dissociation and polymerization of Nrf2 and Keap1 are in a dynamic equilibrium in MRC-5 cells. When MRC-5 cells were stimulated by exposure to radiation, the dissociation of Nrf2 from Keap1 was induced, with subsequent translocation to the nucleus (Figure 5). Therefore, the Nrf2-ARE signaling pathway could protect cells against oxidative stress. Besides, our data show that genistein further increased Nrf-2 translocation to the nucleus after X-ray exposure. Consequently, the expression of Nrf2 downstream genes such as NQO1 and HO1 was up-regulated, and the Nrf2-dependent antioxidant system was enhanced (Figure 5). Because of a strong antioxidant capacity of MRC-5 cells, the induction of ROS by X-rays was suppressed, resulting in an enhanced radioprotective effect on MRC-5 cells.

It has been well established that increased oxidative stress contributes to trigger apoptosis [32], and efflux cytochrome c from mitochondria to the cytosol is essential for Caspase-3 activation and activates the downstream cell death pathway [33]. In the present study, the activation of Caspase-3 was induced by the co-treatment in A549 cells and the cleavage of Caspase-3 was found, as shown in Figure 6B, while the pretreatment with genistein before irradiation obviously limited the specific cleavage of Caspase-3 in MRC-5 cells. Clearly, the correlation between ROS production and mitochondrial cytochrome c release-mediated Caspase-3 activation suggests that the ROS derived from genistein might trigger the apoptosis in A549 cells; however, the combined treatment decreased the apoptotic rate in MRC-5 cells, as shown in Figure 6A. Our study confirms that genistein had a selective radiosensitizing effect on A549 cells, but a significant radioprotective effect on MRC-5 cells.

Many of the radiosensitization drugs are potently cytotoxic to both neoplastic and normal cells. In recent years, targeted therapy has been developed [34]. Our study shows that selective induction of oxidative stress in A549 cells by genistein accounted for its selective radiosensitizing effect, and demethylation of the Keap1 promoter could be the target for genistein. In the study using 47 pairs of NSCLC tissues and normal specimens by Muscarella *et al.*, promoter methylation of Keap1 was detected in 47% of NSCLCs but in none of the normal tissues [35]. Similar results have been obtained in other cancers, including malignant gliomas, breast, colorectal, prostate, thyroid, and head and neck cancer cells [36]. Frequent promoter hypermethylation and correlated down-regulation of the Keap1 expression were observed in malignant gliomas and contributed to the resistance to radiotherapy. So the demethylation effect of genistein on Keap1 may play a broad spectrum role in radiotherapy.

**Figure 7:**
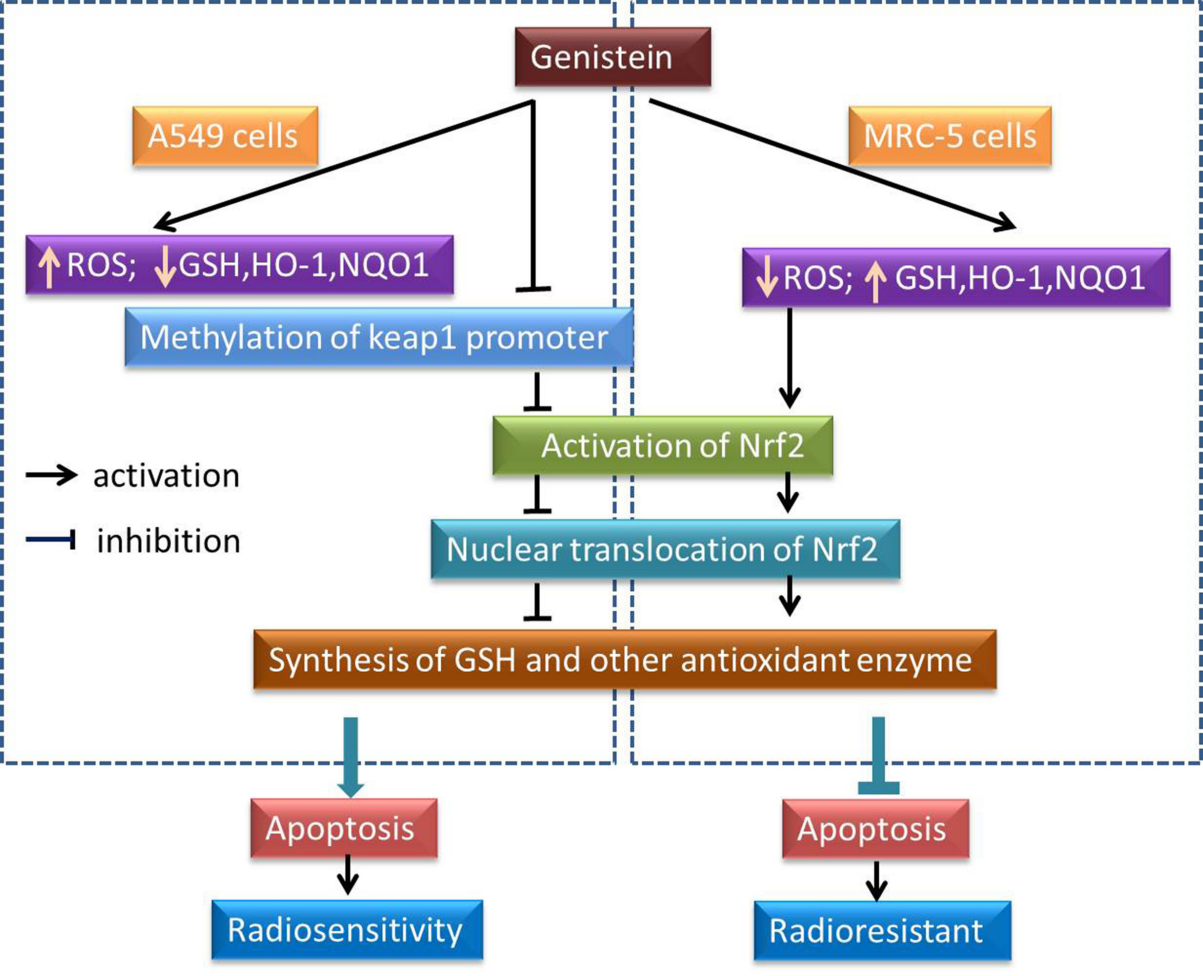
A schematic diagram representing the effect of genistein on the ROS, Nrf2/Keap1 and antioxidant enzymes pathway, which is selective for the radiosensitivity of A549 instead of MRC-5 cells

In sum, our study for the first time shows that differential modulation of the intracellular redox state by genistein in A549 cells and MRC-5 cells was associated with its selective radiosensitizing effect, the corresponding molecular mechanism underlying the differential modulation might be the selective inhibition to methylation of the Keap1 gene promoter region by genistein in A549 cells (Figure 7). Probably, the difference of the redox status between cancerous and normal cells could make it a potential target for radiotherapy, this needs to be further verified using more cell lines.

## MATERIALS AND METHODS

### Cell culture

Human non-small cell lung cancer (NSCLC) A549 cells were cultured in Dulbecco's modified Eagle's medium (DMEM), whereas human normal lung fibroblast MRC-5 cells in Minimum Essential Medium (MEM), with 100 U/mL penicillin, 100 μg/mL streptomycin and 10% fetal bovine serum (Gibco, USA). Cell cultures were performed in a 5% CO_2_ atmosphere at 37°C. Genistein (Sigma, St. Louis, USA) was dissolved in DMSO (Sigma).

### Irradiation

Cells were irradiated with X-rays, which were generated from an X-ray machine (FAXITRON RX-650, Faxitron Bioptics, LLC., Tucson, USA) operated at 50 kVp energy. The dose rate during irradiation was about 0.5 Gy/min. All irradiations were performed at room temperature.

### MTT assay

Cells were seeded in 96-well plates with 5 × 10^3^ cells/well, followed by treatment with DMSO (vehicle) or various concentrations of genistein for 48 h. The MTT assay was performed as the literature described [37].

### Cell growth curve

Cells were treated with 10 μM genistein for 48 h and then were irradiated or sham-irradiated, followed by seeding cells at 5 × 10^3^ cells/well in a 12-well culture plate with a total volume of 2 mL per well. The mean number of cells per well was obtained every day after irradiation from triplicate samples. The data were plotted on a log-linear scale and fitted into an exponential growth curve.

### Colony formation assay

Cells were treated with or without 10 μM genistein for 48 h followed by irradiation or sham-irradiation. Immediately after irradiation, cells were seeded at 200 cells per 60 mm culture dish and cultured for 15 days in fresh medium without genistein, then fixed with alcohol and stained with crystal violet. Colonies containing more than 50 cells were identified as survivors under a stereomicroscope. Each experiment was performed in triplicate.

### Dichlorofluorescein

2 h post-irradiation, cells were washed with serum-free and phenol red-free DMEM, and then loaded with 5 μM 2, 7-dichlorofluorescin diacetate (DCFH-DA). After incubation for 30 min in the dark, the cells were washed with PBS twice and intracellular ROS was measured (excitation, 470 nm; emission, 530 nm, Thermo Varioskan Flash 3001, USA) as reported previously [38].

### Cellular 8-OHdG, PCO and MDA

8-OHdG levels were determined with ELISA using the kit 8-OH-dG-EIA-Biotech (Oxis Health Products Inc., Portland, OR, USA). The carbonyl protein (PCO) and lipid peroxidation MDA were measured with the diagnostic reagent kits (Nanjing Jiancheng Bioengineering, Nanjing, China), respectively. All assays were performed according to the manufacturer's protocol.

### Glutathione (GSH) and glutathione disulfide (GSSG) levels

GSH and GSSG levels were detected with the glutathione kit obtained from Cayman Chemical (Ann Arbor, MI) by following manufacturer's protocol. Briefly, to determine GSSG levels, GSH was masked by 2-vinyl pyredine for 1 h before the assay. The samples were read at 405 nm at 5 min intervals for 30 min. The GSH and GSSG levels were evaluated by comparison with standards and normalized with protein content. The results were expressed as total GSH or GSH/GSSG ratio, using reduced form GSH or an oxidized form of GSH (GSSG) as the standard.

### Western blot

Western blot analysis was performed as previously described [39] using corresponding antibodies against Nrf2, NQO1, HO-1, Caspase-3, Cytochrome c, Lamin B1 (Cell Signaling Technology, Boston, USA), GAPDH (Sigma).

### Reverse transcriptase-polymerase chain reaction (RT-PCR)

Reverse transcription for cDNA synthesis was performed on 2 μg total RNA using a Transcriptor First Strand cDNA Synthesis Kit (Roche, Mannheim, Germany) with anchored-oligo(dT)_18_ primers. The housekeeping gene b-actin served as an internal control to confirm the success of the reverse transcription reaction. The PCR products were subjected to 1.5% agarose gel electrophoresis. Primer sequences were as follows: Keap1 forward: 5′-GACAGCCTCTGACAACACAAC-3′, reverse: 5′-GAA ATCAAAGAACCTGTGGC-3′; b-actin forward: 5′-GGAA ATCGTGCGTGACATTA-3′, reverse: 5′-GGAGCAATG ATCTTGATCTTC-3′ [17]. PCR cycling conditions were 94°C (3 min) for one cycle, 94°C (30 s), 55°C (45 s), and 72°C (1 min) for 30 cycles, and a final extension of 72°C (5 min).

### 5-Aza-2′-deoxycytidine treatment

5-Aza-2′-deoxycytidine (5-Aza, Sigma, St. Louis, MO, USA) was dissolved in phosphate-buffered saline (PBS). Exponentially growing cells were incubated in culture medium with and without 5-Aza at a concentration of 10 μM for 5 days, with the medium changed daily. After cells were harvested, RNA and DNA were extracted for RT-PCR and DNA methylation analysis, respectively.

### Bisulfite pyrosequencing

50 ng of bisulfite-treated genomic DNA was amplified in an Opticon II system (MJ Research, MA, USA) using the HS taq PCR kit (Takara, Otsu Shiga, Japan). PCR amplification primers for the Keap1 promoter region were as follows: forward: 5′-GTTTGAGGTTAGGAGTTTAAGGTTG-3′, reverse: 5′-CACAACCAAACCCCCCTT-3′. The reverse primer contained biotin at the 5′ position. Two assays were designed and run on this template using two sequencing primers: 5′-GAGGTAGATGATTTTTTTTAGAT-3′ (assay for CpGs 1-7) and TAAAAGGAGAATAGTAGATGGTG (assay for CpGs 8–13). For the pyrosequencing reaction, single-stranded DNA templates were immobilized on streptavidin-coated sepharose beads (Qiagen, Hilden, Germany) using the PSQ Vacuum Prep Tool and Vacuum Prep Worktable (Qiagen, Hilden, Germany), according to the manufacturer instructions, then incubated at 80°C for 2 min. Pyrosequencing was performed using PyroMark Q24 (Qiagen, Hilden, Germany).

### Apoptosis

Apoptosis was quantified by means of a combined staining of Annexin V and PI using the Annexin V-FITC Apoptosis Detection Kit (BestBio, Shanghai, China). The specific method was the same as described previously [39].

### Statistical analysis

Statistical analysis was performed using either the Student's *t* test (for two-group comparison) or the one-way ANOVA (for multiple-group comparison). Data are represented as mean ± S.E.M.
